# Alpha-Synuclein Fails to Form Aggregates in Endocytosis-Defective Fission Yeast Strains, ∆ *myo1* and ∆ *end4*

**DOI:** 10.17912/micropub.biology.001479

**Published:** 2025-01-21

**Authors:** Teruaki Takasaki, Ryuga Yamada, Yoshitaka Sugimoto, Reiko Sugiura

**Affiliations:** 1 Faculty of Pharmacy, Kindai University

## Abstract

Alpha-Synuclein (α-Syn) is a soluble neuronal protein whose aggregation is one of the hallmarks of Parkinson's disease (PD). We previously developed a fission yeast model of PD that recapitulates α-Syn aggregation upon high-level expression of human α-Syn. Here, we show that α-Syn aggregate formation in yeast requires Myo1 and End4 , proteins essential for the early steps of endocytosis. α-Syn expression levels in Δ *myo1* and *∆end4* cells were comparable to wild-type cells, suggesting that defects in endocytosis disrupt α-Syn aggregation. These findings highlight the critical role of endocytosis in α-Syn aggregation and PD pathology.

**Figure 1. Overexpression of α-Syn leads to aggregate formation in WT cells, but not in Δ myo1 and ∆end4 cells.**
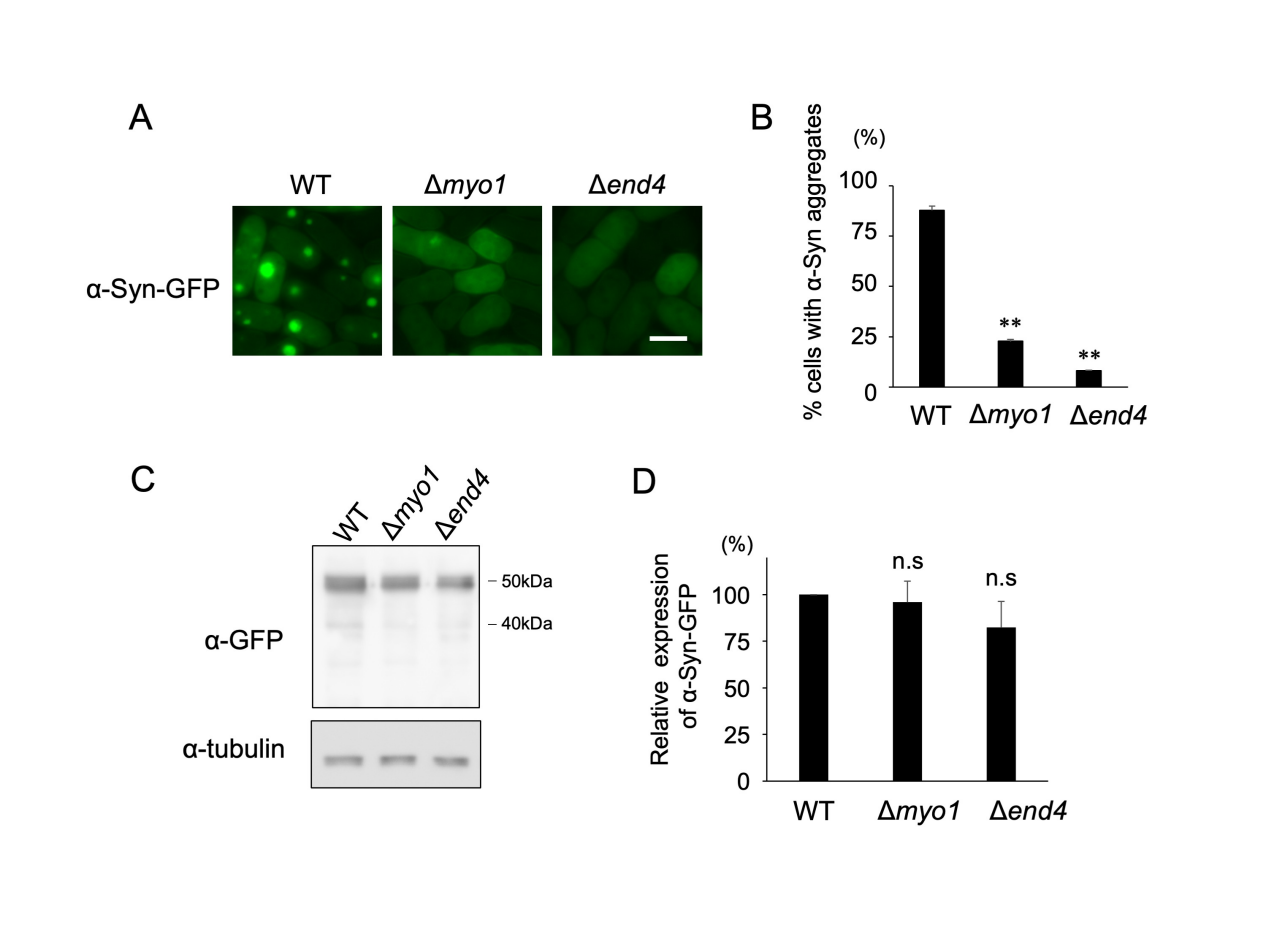
**A:** Representative images of the wild-type (WT), Δ *myo1* and *∆end4* cells transformed with the plasmid containing α-Syn-GFP gene under the *adh1* promoter. Cells were incubated at 27˚C for 18 h. Scale bar: 5 µm **B:** Percent cells with α-Syn-GFP aggregates in the cytoplasm are shown. More than 50 cells were counted for each independent experiment. Graphs show mean ± S.D. (n=2). **P<0.01. **C:** Protein expression levels of α-Syn-GFP were analyzed by immunoblotting with anti-GFP and anti-tubulin antibodies. **D:** Relative expression levels of α-Syn were measured as the ratio between the intensities of the α-Syn-GFP and tubulin, and normalized to WT cells. Graphs show mean ± S.D. (n=3). n.s.: not significant.

## Description

Alpha-synuclein (α-Syn) is a small 140-amino acid protein that is abundant in human brain (Maroteaux et al. 1988; Burré et al. 2018) . Under physiological conditions, α-Syn is intrinsically disordered and soluble; however, postmortem samples from the basal ganglia and limbic cortex of Parkinson's disease patients show increased insoluble α-Syn compared to age-matched controls (Mamais et al. 2013) . Aggregation of α-Syn has been implicated in the pathogenesis of several neurodegenerative disorders, including Parkinson's disease and dementia with Lewy bodies, collectively termed synucleinopathies (Goedert 2001; Srinivasan et al. 2021) . However, the mechanisms that induce α-Syn aggregation are not fully elucidated.

Previously, we have generated a fission yeast Parkinson's disease model that recapitulates α-Syn aggregate formation by forcing the cells to express human α-Syn at high levels (Sugimoto et al. 2025) . Using this yeast model, we found that rapamycin and Torin1, inhibitors of mammalian target of rapamycin (mTOR), potently inhibit the formation of α-Syn aggregates without altering α-Syn expression (Sugimoto et al. 2025) . This discovery prompted us to hypothesize that mTOR function(s) other than regulation of protein synthesis is required for α-Syn aggregate formation. mTOR is a conserved protein kinase that regulates a variety of fundamental cellular processes, including protein synthesis, nutrient sensing, autophagy, endocytosis, and cell proliferation (Grahammer et al. 2017; Goul et al. 2023; Panwar et al. 2023) . Among mTOR's many roles, in this study, we directly tested whether endocytosis is involved in α-Syn aggregate formation using a genetic approach.

We first confirmed that overexpression of the α-Syn-GFP fusion protein leads to aggregate formation in wild-type cells, as reported in a previous study (Sugimoto et al. 2025) ( Fig. 1A ). Approximately 80% of the cells exhibited cytoplasmic α-Syn aggregates ( Fig. 1B ). Next, we overexpressed the α-Syn-GFP fusion protein in the cells lacking *myo1* ^+^ gene (∆ *myo1* ). *myo1* ^+^ encodes a type I myosin that regulates the actin assembly/disassembly process and is required for the early steps of endocytosis (Lee et al. 2000; Toya et al. 2001; Petrini et al. 2015) . Notably, α-Syn-GFP failed to form aggregates in ∆ *myo1* cells ( Fig. 1A, B ). We also tested whether similar defects in the α-Syn aggregate formation were observed in the cells lacking *end4* ^+^ , which is essential for the internalization process of the endocytic pathway, but not for the later stages of endocytosis, such as vacuolar protein transport (Iwaki et al. 2004) . As we predicted, α-Syn aggregates were rarely detected in ∆ *end4* cells ( Fig. 1A, B ).

Given that the expression levels of α-Syn-GFP in ∆ *myo1* and ∆ *end4* cells were comparable to those in wild-type cells ( Fig. 1C, D ), the failure in α-Syn aggregate formation is likely due to defects in the early step of endocytosis. The early step of endocytosis involves the internalization of a portion of the plasma membrane to incorporate membrane bound proteins, lipids, and extracellular components (Kumari et al. 2010) . As α-Syn inclusions are known to contain a high amount of lipid membrane (Shahmoradian et al. 2019) , the step of internalizing cell membrane components might be required to provide a scaffold to form α-Syn aggregates. Alternatively, the process of endocytosis may contribute more directly to the α-Syn aggregate formation, for example, by internalizing and accumulating membrane-bound α-Syn through the endocytic pathways. Our findings contribute to a deeper understanding of the mechanisms underlying the α-Syn aggregate formation and the Parkinson's disease pathology.

## Methods

Standard yeast culture and genetic methods were used except where otherwise noted (Sabatinos and Forsburg 2010) . Yeast strains harboring the plasmid pKD4557 [ *P adh1* - *α-syn-GFP* ] (Sugimoto et al. 2025) were grown in Edinburgh minimal medium (EMM) in conical tubes at 27°C for 18 h. Images were acquired by an All-in-one fluorescence microscope (BZ-X710, KEYENCE, Osaka, Japan).

## Reagents

**Table:** 

Strain	Genotype	Reference
HM123	* h ^−^ leu1-32 *	Lab stock
KP1257	* h ^−^ leu1 myo1 ::kanr *	Ma et al. 2011
KP4150	* h ^−^ leu1 ura4 arg1 end4::ura4 ^+^*	Iwaki et al. 2004
